# Nephroprotective effects of Candesartan Cilexetil against Cyclosporine A-induced nephrotoxicity in a rat model

**DOI:** 10.25122/jml-2021-0227

**Published:** 2022-12

**Authors:** Fadhaa Abdulameer Ghafil, Samah Abdulridha Abdul Kadhim, Sahar Majeed, Heider Qassam, Najah Rayish Hadi

**Affiliations:** 1 Department of Pharmacology and Therapeutics, Faculty of Medicine, University of Kufa, Najaf, Iraq; 2 Al-Diwanyah Health Directorate, Al-Diwanyah, Iraq

**Keywords:** Cyclosporine A (CsA), Candesartan Cilexetil (CC), nephrotoxicity, CC – Candesartan Cilexetil, CAT – catalase, COX2 – Cyclooxygenase-2, CsA – cyclosporin A, GFR – glomerular filtration rate, Gpx1 – glutathione peroxidase 1, IL-2 – interleukin-2, MDA – malondialdehyde, NFκB – Nuclear factor kappa B, ROS – reactive oxygen species, RAAS – renin-angiotensin-aldosterone system, SOD – superoxide dismutase, TGF-ß1 – transforming growth factor beta 1, VEGF – vascular endothelial growth factor

## Abstract

Cyclosporine A (CsA), a well-known immunosuppressive drug, has been prescribed after organ transplantation and in a variety of disorders with an immunological origin. Nephrotoxicity is one of the most frequently stated problems associated with CsA, and therefore the treatment with CsA remains a big challenge. This study sets out to assess the ameliorative influences of Candesartan Cilexetil (CC) on oxidative stress and the nephrotoxic effect of CsA in a rat model. Twenty-four Wister Albino rats, 7–8-week-old, weighing 150–250g, were randomly categorized into three groups (eight animals in each group). These groups were the (1) CsA-treated group, (2) vehicle-treated group, and (3) CC-treated group. Bodyweights were assessed at the start and end of experiments. Renal function test and levels of glutathione peroxidase 1 catalase -CAT (Gpx1), catalase (CAT), superoxide dismutase (SOD), interleukin -2 (IL-2), and malondialdehyde (MDA) were investigated in renal tissues. Histological changes in kidneys were also evaluated. Data showed that levels of urea and creatinine in serum and levels of IL-2 and MDA in renal tissues were elevated in the CsA-treated group, with severe histological changes compared with the control group. Furthermore, tissue levels of Gpx1, CAT, and SOD were significantly decreased in CsA-treated in comparison with the control group. Treatment with CC for the rats subjected to CSA resulted in a marked reduction in levels of serum urea and creatinine and tissue levels of IL-2 and MDA. Levels of Gpx1, CAT, and SOD in renal tissues were greater in the CC-treatment group compared with the CsA-treated group. CC treatment reduced the deterioration of renal morphology compared with CsA treatment. The findings of this study suggest that CC could prevent CSA-induced nephrotoxicity through its anti-inflammatory and antioxidant influences. Considerably more work needs to be done to determine the mechanistic insight behind the ameliorative effect of CC.

## INTRODUCTION

Cyclosporine A (CsA) is one of the main calcineurin inhibitor drugs (drugs that impair the immune response) available for clinical use to reduce the activity or efficacy of the immune system, designed to treat certain disorders, such as autoimmune illnesses, or to decrease the ability of the body to reject a transplanted organ [1]. Nephrotoxicity, neurotoxicity, and other adverse effects limit the use of CsA, thereby, special attention is paid to reducing these unwanted effects [2]. Very little is currently known about the molecular mechanisms that underpin the effect of CsA in kidneys. Several studies highlighted the role of oxidative stress and the renin-angiotensin-aldosterone system (RAAS) in the pathogenesis of nephrotoxicity [3, 4]. Moreover, intrarenal activation of RAAS is a critical factor in the pathogenesis of chronic CsA nephrotoxicity [5]. The RAAS is a potent vasoactive factor resulting in vasoconstriction and thus leading to ischemia [6]. CsA activates RAAS through its direct effects on juxtaglomerular cells [7, 8]. Cyclooxygenase-2 (COX-2) and renin synthesis have been found to be activated by CsA treatment, leading to a reduction in sodium and water excretion as well as glomerular filtration rate (GFR) [7]. RAAS has been reported to increase renal damage by its influences on a variety of factors such as activation of vascular endothelial growth factor (VEGF), transforming growth factor beta 1 (TGF-ß1), inflammation, and apoptosis [6]. Several reports highlighted the detrimental effect of CsA on renal tissues through modulating a variety of molecules and processes, for instance, the increased levels of thromboxane and reactive oxygen species (ROS) and lipid peroxidation. Furthermore, CsA resulted in marked changes in the activity and expression levels of several enzymes such as COX-2, catalase (CAT), superoxide dismutase (SOD), glutathione peroxidase (Gpx1) [9, 10]. It has been reported that treatment with CsA increased levels of free radicals in urine [11]. Candesartan Cilexetil (CC), belonging to the angiotensin receptor blockers family, is widely prescribed for patients with hypertension [12]. This family has anti-inflammatory effects via inhibition of angiotensin II, a proinflammatory molecule [13]. The nuclear factor kappa B (NFκB) signaling pathway has been shown to be involved in angiotensin II-induced cytokines activation and to initiate the production and release of different molecules such as chemokines and adhesion molecules [14]. Antagonizing the AT1 receptor has been found to decrease the synthesis of superoxide anions by reducing the activity of angiotensin II in endothelium and vascular smooth muscles [15]. This, in turn, protects the nitric oxide from converting into detrimental molecules [16]. AT1 receptor blocker family has been reported to modulate the oxidative stress molecules, leading to improvements in markers of kidney disease [17–20].

## MATERIAL AND METHODS

### Ethical statement and animals maintenance

Seven to eight-week-old Albino Wistar rats weighing between 150 and 250 g were obtained from the Faculty of Sciences, University of Kufa, Iraq. All animals were housed in the laboratory animals house at the University of Kufa. Animals were maintained at a temperature of 22±2℃ in a 12h light/dark cycle and allowed access to water and food.

### Experimental protocol

Animals were assigned into three groups, each group containing eight animals. The vehicle-treated group was given normal saline by gavage tube at a dose of 4 ml/kg/day and olive oil subcutaneously injected at a dose of 2 ml/kg/day. The CsA-treated group was subcutaneously injected with CsA (20 mg/kg/day dissolved in olive oil), and normal saline was given by intragastric gavage [21]. The CC-treated group was given CC using a gavage tube (1 mg/kg/day, dissolved in normal saline) 1 day before the CsA injection. All experimental groups received the intervention for three weeks.

### Drugs

CsA was purchased from Cheme Scene, USA, Cat. (No. CS-2761). The CsA was prepared by dissolving it in olive oil and administering the solution via a subcutaneous route at a dose of 2 mg/kg/day [22, 23]. CC obtained from AstraZeneca was dissolved in normal saline and given via gavage tube at a dose of 1 mg/kg/day [24].

### Preparation of samples

On the 22^nd^ day of the experiment, animals were intraperitoneally injected with xylazine (10 mg/kg) and ketamine (100 mg/kg) [25]. The serums were obtained by collecting the blood from the heart and allowing the blood to clot at room temperature for 30 min. The samples were then centrifuged for 10 min at 3000 rpm. The supernatants were used to measure the serum levels of urea and creatinine [26].

#### Measurement of the levels of CAT, SOD, MDA, GPx1, and IL-2 in the renal tissues using ELISA

On the 22^nd^ day of the experiment and after the animals were sacrificed, the right kidneys were removed and washed with normal saline. Each kidney was then weighted. Further on, phosphate buffer containing 1% Triton X-100 and protease inhibitor cocktail was added in a ratio of 1:10 w/v. The samples were then homogenized using a high-intensity liquid processor [27]. The homogenates were spun down at 10000 rpm for 10 min at 4℃. Supernatants were used for measuring the levels of the CAT, SOD, malondialdehyde (MDA), GpX1, and interleukin -2 (IL-2).

#### Preparation of tissue samples for Histopathology

For histological evaluation, the left kidneys were removed on the 22^nd^ day and put in a 10% formalin. Tissue samples were paraffinized and then cut into slices of 5 µM in thickness [28]. The slices were deparaffinized and stained with hematoxylin and eosin [29]. The tissue sections were visualized under a bench microscope. The percentage of tissue damage was assessed using scores ranging from 0 to 4 [30], as seen in Table 1.

**Table 1:** Percentage of tissue damage.

Score	Percentage of tissue damage
**0**	No damage
**1**	≤25% of the tubules
**2**	25–50% of the tubules
**3**	51–75% of the tubules
**4**	76–100% of the tubules

### Statistical analysis

All results were analyzed using the SPSS software version 20. Data were presented as mean±SD unless otherwise stated. To compare the parameters between the study groups, a one-way ANOVA was used, followed by a posthoc test. In addition, bodyweight differences at the start and end of the experiment were analyzed using paired t-tests. A P-value≤0.05 was considered statistically significant.

## RESULTS

### Effect of CC treatment on indicators of renal damage

**Figure 1:**
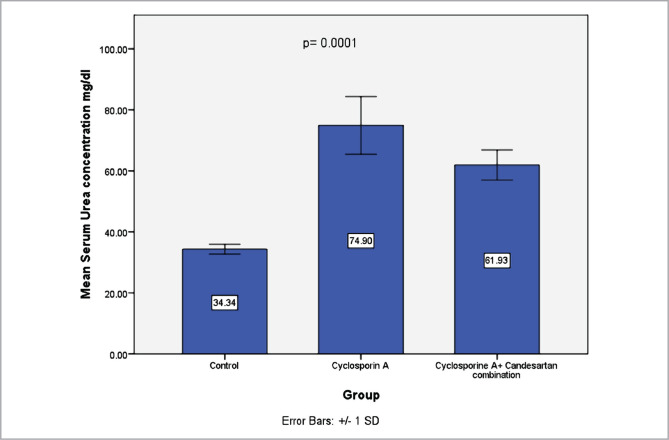
Mean serum levels of urea (mg/dl) in three experimental groups. Results are expressed as mean±SD, n=8.

**Figure 2:**
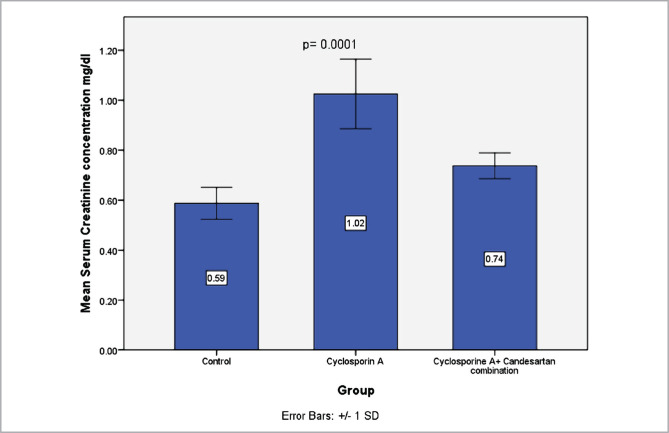
The mean serum concentrations of creatinine (mg/dl) in three experimental groups. Data were presented as mean±SD, n=8.

Serum levels of urea and creatinine were evaluated to investigate the function of the kidneys. Data revealed that levels of these indicators increased markedly in the CsA-treated group compared to the control group. However, these levels were significantly decreased in the CC-treated group (Figures 1 and 2).

### Effect of CC treatment on the levels of MDA in renal tissues

**Figure 3:**
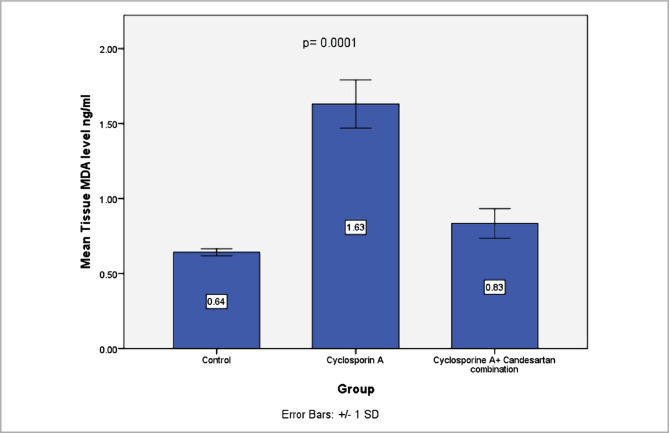
Mean levels of MDA (ng/ml) in renal tissues in three experimental groups. Data are expressed as mean±SD, n=8.

MDA levels, an indicator of lipid peroxidation, were assessed in renal tissues using an ELISA assay. Data showed that MDA levels were elevated in the CsA-treated group *versus* the control group. CC treatment resulted in a marked decrease in levels of MDA (Figure 3).

### Effect of CC treatment on levels of SOD in renal tissues

**Figure 4:**
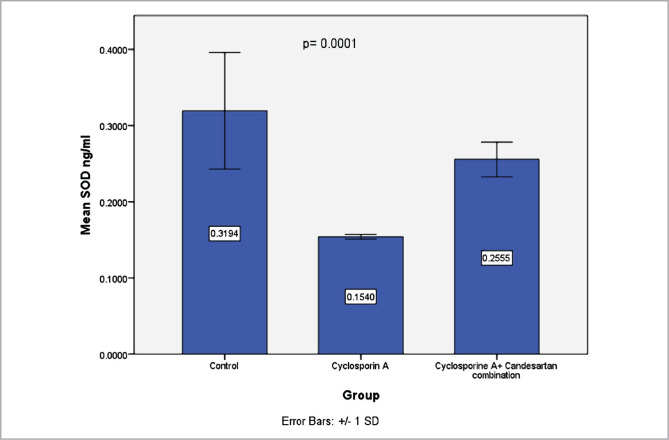
Mean levels of SOD (ng/ml) in three experimental groups. Data are presented as mean±SD, n=8.

SOD, a biomarker of oxidative stress, was investigated. Data revealed that SOD levels were lower in the CsA group than in the control group. Compared to the CsA group, the group on the CC treatment showed a marked increase in levels of SOD (Figure 4).

### Effect of CC treatment on levels of CAT in renal tissues

**Figure 5:**
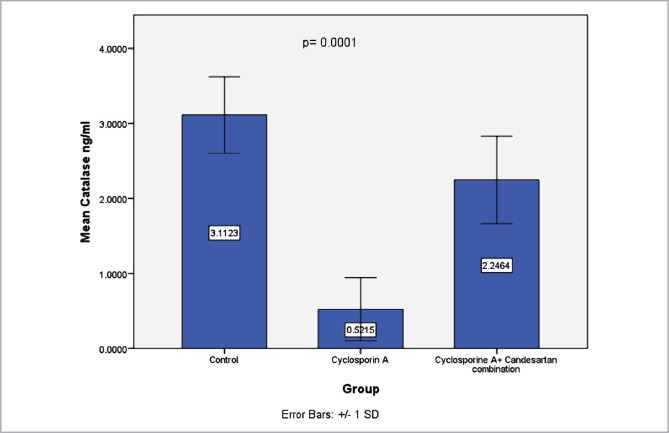
Mean levels of CAT (ng/ml) in three experimental groups. Data are presented as mean±SD, n=8.

Data revealed that levels of CAT in the CsA group were lower than in the control group. However, in contrast to earlier findings, CC treatment resulted in a marked elevation in the levels of CAT in comparison with the CsA group (Figure 5).

### Effect of CC treatment on levels of GpX1 in renal tissues

**Figure 6:**
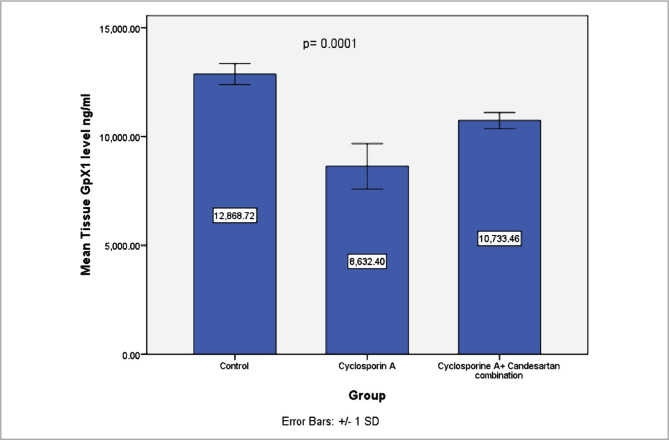
Mean levels of GpX1 (ng/ml) in renal tissues in three experimental groups. Data were presented as mean±SD, n=8.

Levels of GpX1 were measured to investigate the potential antioxidant effect of CC. The results revealed that levels of GpX1 in renal tissues decreased significantly in the CsA group compared to the control group. However, CC treatment increased levels of GpX1 in comparison with the CsA group (Figure 6).

### Effect of CC treatment on levels of IL-2 in renal tissues

**Figure 7:**
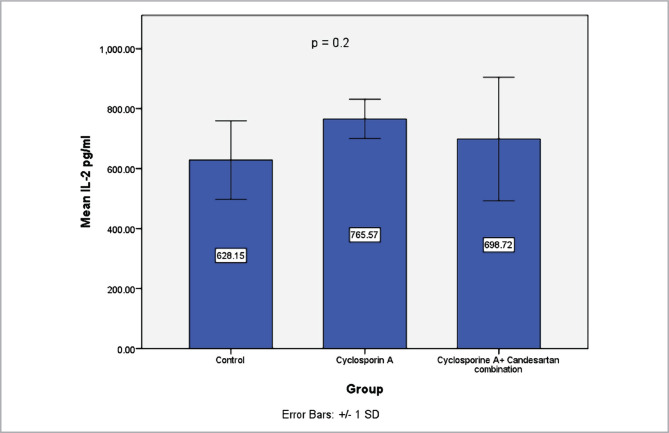
Mean levels of IL-2 (pg/ml) in renal tissues in three experimental groups. Data are expressed as mean±SD, n=8.

Data revealed a marked elevation in the levels of IL-2 in the CsA group *versus* the control group. These increments were significantly decreased in the CC treatment group (Figure 7).

### Effect of CC treatment on body weight

Paired T-tests were used to compare the difference in body weight between the start and end of the experiment. They showed that bodyweights in the CsA group decreased markedly at the end of experiments as compared to bodyweights at the start. By contrast, the body weights of the CC-treated group increased at the end of experiments compared to the start of experiments (Table 2).

**Table 2:** Mean bodyweights (grams) among three study groups at the start and end of the experiments. Data are presented as mean±SD, n=8.

Group	Mean bodyweights (grams) at the start of experiments	Mean bodyweights (grams) at the end of experiments	P-value
**Control**	166.3750±7.8	188.3750±16.1	0.0001
**CsA**	168±17.7	158.3±20.9	0.0001
**CsA+Candesartan**	170.4±16	177.3±12.6	0.001

### Histopathological evaluation

**Figure 8:**
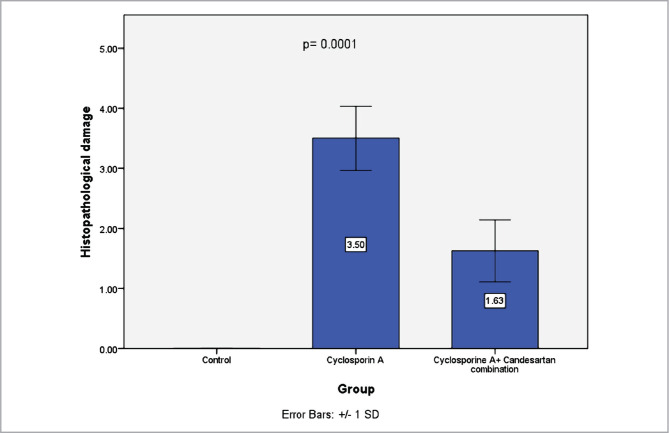
Mean scores of severities of tissue damage among the three study groups. Data are presented as mean±SD, n=8.

Histological data showed that the CsA group had a higher degree of renal tissue damage compared to the control group, with values represented as mean±SD (Figure 8). Conversely, CC treatment resulted in a marked amelioration in the renal tissues compared with the CsA group (Figure 8).

**Figure 9:**
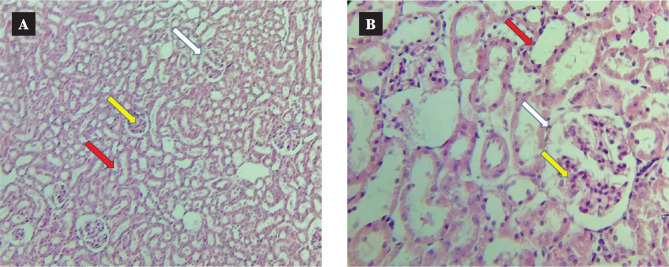
The normal renal tissues (red arrow), normal glomeruli (white arrow), and normal capillary tuft (yellow arrow) in the control group. A: 10×10 magnification. B: 10×40 magnification.

**Figure 10:**
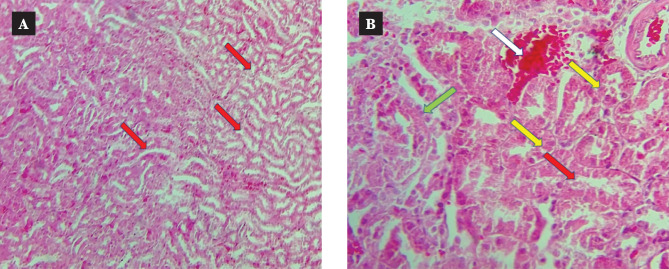
Cross tissue section stained with hematoxylin and eosin, characterized by a marked diffuse in the renal tubules (red arrow) (10×10 magnification). B: The image revealed cellular swelling (indicated in yellow arrow), Eosinophilic cytoplasm (indicated in green arrow), and tubular dilation (indicated in red arrow) (10×40 magnification).

**Figure 11:**
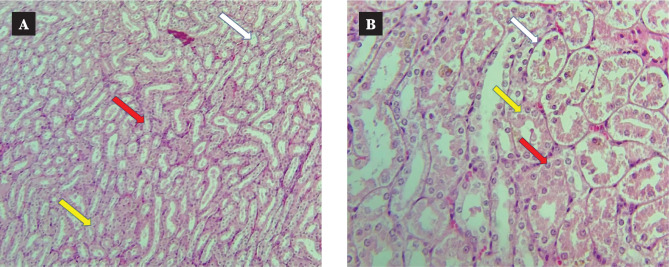
Sections of the renal tissues stained with hematoxylin and eosin. A: The CC treatment group showed a marked improvement with normal tubular cells (white arrow) and mild edema (yellow and red arrows) (10×10 magnification). B: CC treatment revealed a 3% of tubular cell damage (white and yellow arrows) and indicated by cellular swelling and Eosinophilic cytoplasm (red arrow) (10×40 magnification).

Representative images revealed that the control group presents normal tissue sections (Figure 9 A, B) [31]. In contrast, the CsA group had noticeable damage in different locations, such as renal tubules, glomeruli, and capillary tuft (Figure 10 A, B) [31]. CC treatment resulted in a marked amelioration in renal tissue sections compared with the CsA group (Figure 11 A, B).

## DISCUSSION

Cyclosporine A is an immunosuppressive drug used in organ transplantation to prevent rejection, where it significantly decreases morbidity and mortality [32]. Unfortunately, cyclosporine toxicity which is attributed in part to reactive oxygen species and oxidative stress, may limit its use [3].

The current study shows that treatment with CsA resulted in a deterioration in renal function by increased serum levels of urea and creatinine compared with the control group. These data match those observed in earlier studies, "Effect of CsA on study parameters" [33, 34].

This result may be explained by the fact that renal vasoconstriction is a critical player in the pathogenesis of renal injury. This can be aggravated by a variety of molecules such as angiotensin II and vasoactive substances [23]. Moreover, renal function can be deteriorated by oxidative stress, which is a key player in inducing many vasoactive mediators resulting in vasoconstriction and leading to reduced GFR [35, 36].

The present study reveals that levels of MDA in the renal tissue increased significantly in CsA groups compared to the control group. These results are in line with those of previous studies [36]. These data are likely related to increased lipid peroxidation [37]. It has been reported that increments in free radicals, lipid peroxides, and MDA were found after treatment with CsA [38]. Furthermore, CsA treatment resulted in a marked elevation in hydrogen peroxide, superoxide, and hydroxyl radical resulting in lipid peroxidation and nephrotoxicity [31].

The current study found that levels of SOD in the CsA group decreased compared to the control group. This finding is in line with other studies in which it was discovered that chronic treatment of rats with CsA resulted in a marked reduction in the levels of SOD, suggesting increased lipid peroxidation with an increment in oxidative stress load and reduced antioxidant capacity [32, 33]. SOD is a powerful endogenous antioxidant mopping the free radicals that are detrimental to the cells [33]. Low levels of SOD in the renal tissue have been found under CSA treatment which suggests increased oxidative load [39].

The present study shows that levels of GpX1 in the renal tissue decreased markedly in the CsA group *versus* the control group, consistent with previous research [31–33]. CsA treatment-induced GpX1 reduction could be an indicator of the low levels of glutathione and high levels of peroxide. Decreased levels of glutathione can lead to low antioxidant capacity and increased free radicals production [38].

The results of this study indicate that CAT levels in renal tissues increased in CsA group compared to the control group, consistent with other research [32]. It seems possible that these results are due to a drop in the levels of NADPH that mediates the activity of CAT [38].

There are several possible explanations behind the decreased levels of SOD, CAT, and GpX1 in the renal tissues after CsA treatment. First, CsA treatment may result in hypoxia and reoxygenation via an imbalance between vasoconstriction and vasodilation, leading to a generation of ROS in the renal tissue [40]. Second, CsA treatment could disrupt mitochondrial respiration resulting in a free radical generation [34]. Third, it could modulate a variety of enzymes in the kidney, such as SOD, CAT, and GpX1 [10]. It has been reported that CsA treatment resulted in a marked increase in free radicals in urine, suggesting their effect on the urinary system [11].

The current study shows elevated levels of IL-2 in the CsA group compared to the control group. IL-2 increases in response to a variety of pathophysiological stimuli [41]. The increased levels of IL-2 in the CsA group could be due to oxidative stress leading to renal dysfunction. Activation of RAS could be mediated by excessive production of TGFß that is induced by CsA treatment [1]. Nuclear factor kappa B (NF-Kß) is a critical factor in mediating the activation of cytokines via angiotensin II increasing the production of cytokines, chemokine, and adhesion molecules [14]. RAS system has been found to induce renal injury via stimulation of the Tubulointerstitial inflammation [6]. The finding of this study showed that bodyweights at the end of the experiment decreased compared to the start of the experiment in the CsA group. These results are in line with those of previous studies [31–34]. A possible explanation for this might be the oxidative stress that could be responsible for decreased body weight [3]. Histological evaluation showed that renal tissues of the CsA group deteriorated and changed in morphology compared with the control group. These results match those observed earlier [35–37]. Although the mechanism associated with renal injury after CsA treatment remains incompletely understood, several suggestions are reported with respect to the role of RAAS [4], including increased synthesis and release of angiotensin II, increased production endothelin, changes in thromboxane and prostanoid synthesis, impaired in nitric oxide production and alteration in gene expression, and these could be ended to nephrotoxicity [3].

### Influence of CC treatment on CsA–induced renal injury

The current study shows that treatment with CC in rats exposed to CsA treatment resulted in a marked reduction in levels of serum urea and creatinine compared with the CsA group. This finding is consistent with an earlier report [24]. It has been outlined that AT1 receptor antagonists have a role in reducing oxidative stress, thereby ameliorating renal tissues [18]. The pathogenesis tends to be linked to renal vasoconstriction induced by angiotensin II and other vasoactive molecules [23]. AT1 receptor antagonist can inhibit the action of Angiotensin II [12]. The present study reveals that levels of MDA in the renal tissues decreased in the CC treatment group in comparison with the CsA group. The findings of our research agree with those obtained previously [24].

These results are likely related to the potential antioxidant effect of CC that could reduce lipid peroxidation and MDA levels. Furthermore, it has been shown that the AT1 receptor blockers family reduces levels of oxidative stress in the renal tissues [17]. The current study shows that levels of SOD, GpX1 and CAT increased markedly in CC treatment groups compared to the CsA group, consistent with the previous report [24]. Superoxide anions generation decreases after treatment with an AT1 receptor antagonist, and this could be due to inhibition in the activity of angiotensin II-dependent oxidase in the vascular smooth muscle and endothelial cells [15]. In addition, AT1 receptor antagonists have been found to decrease oxidative stress in several studies [18].

The present study shows that body weights at the end of the experiments increased compared with the start in the CC treatment group. This result may be explained by the fact that CC could have antioxidant and anti-inflammatory properties that protect the kidney from being damaged by CsA treatment. The present study revealed that CC treatment ameliorates renal morphology compared with the CsA group, consistent with the previous report [33].

It seems possible that these results are due to the anti-inflammatory and antioxidant influences of CC. Inhibiting the proinflammatory impacts of angiotensin II is the possible mechanism by which the AT1 receptor antagonists can improve renal morphology [13].

## CONCLUSION

This study shows that Candesartan Cilexetil could protect the renal tissues from being damaged by CsA treatment, and these findings could be supported by the Candesartan Cilexetil effects on oxidative stress and proinflammatory markers.
